# EDUCATION, RACE-ETHNICITY, AND MULTI-MORBIDITY AMONG ADULTS AGES 30-64 IN THE NATIONAL HEALTH INTERVIEW STUDY

**DOI:** 10.1093/geroni/igz038.1282

**Published:** 2019-11-08

**Authors:** Vicki Johnson-Lawrence, Anna Zajacova, Rodlescia Sneed

**Affiliations:** 1 Michigan State University, Flint, Michigan, United States; 2 University of Western Ontario, London, Ontario, Canada

## Abstract

Demographic risk factors for multimorbidity (living with 2+ chronic conditions) have been identified in numerous population-based studies of older adults; however, there is less data on younger populations, despite the fact that approximately 24% of US adults age 18+ have multimorbidity. To examine the associations of education and race/ethnicity with mutimorbidity among adults aged 30-64 using cross-sectional data from the 2002-2014 National Health Interview Surveys. Compared to having a bachelor’s degree or higher, completing less than HS (OR=1.58, 95% CI = 1.50-1.66) or HS/some college (OR=1.32, 95% CI = 1.27-1.37) were both associated with increased odds of multimorbidity. Non-Hispanic Blacks had greater odds of multimorbidity (OR=1.07, 95% CI = 1.02-1.11) compared to Non-Hispanic Whites with comparable characteristics. Reducing multimorbidity through health promotion efforts across the socioeconomic spectrum and earlier in the life course will be a requirement to age successfully and support overall well-being in the aging US population.

